# Deep learning tools are top performers in long non-coding RNA prediction

**DOI:** 10.1093/bfgp/elab045

**Published:** 2022-02-06

**Authors:** Tea Ammunét, Ning Wang, Sofia Khan, Laura L Elo

**Affiliations:** Turku Bioscience Centre, University of Turku and Åbo Akademi University, Turku, Finland; Turku Bioscience Centre, University of Turku and Åbo Akademi University, Turku, Finland; Turku Bioscience Centre, University of Turku and Åbo Akademi University, Turku, Finland; Turku Bioscience Centre, University of Turku and Åbo Akademi University, Turku, Finland; Institute of Biomedicine, University of Turku, Turku, Finland

**Keywords:** lncRNA, benchmark, machine learning

## Abstract

The increasing amount of transcriptomic data has brought to light vast numbers of potential novel RNA transcripts. Accurately distinguishing novel long non-coding RNAs (lncRNAs) from protein-coding messenger RNAs (mRNAs) has challenged bioinformatic tool developers. Most recently, tools implementing deep learning architectures have been developed for this task, with the potential of discovering sequence features and their interactions still not surfaced in current knowledge. We compared the performance of deep learning tools with other predictive tools that are currently used in lncRNA coding potential prediction. A total of 15 tools representing the variety of available methods were investigated. In addition to known annotated transcripts, we also evaluated the use of the tools in actual studies with real-life data. The robustness and scalability of the tools’ performance was tested with varying sized test sets and test sets with different proportions of lncRNAs and mRNAs. In addition, the ease-of-use for each tested tool was scored. Deep learning tools were top performers in most metrics and labelled transcripts similarly with each other in the real-life dataset. However, the proportion of lncRNAs and mRNAs in the test sets affected the performance of all tools. Computational resources were utilized differently between the top-ranking tools, thus the nature of the study may affect the decision of choosing one well-performing tool over another. Nonetheless, the results suggest favouring the novel deep learning tools over other tools currently in broad use.

## Introduction

Accurately defining protein-coding transcripts from non-coding transcripts is of increasing importance, as large numbers of non-coding RNA transcripts have been discovered in the human genome [1, 2]. Early findings of key long non-coding RNAs (lncRNA), advances in technology and the completion of the human genome have prompted the investigation of all transcribed regions of the genome, leading to the discovery of many lncRNA molecules with significance in healthy and diseased cell functioning [3]. However, many factors, from novel splice-site detection to transcript assembly, create difficulties in obtaining a robust set of candidate non-coding transcripts from sequence data (reviewed in for example [4]). Therefore, a myriad of bioinformatics tools has been developed solely to address the question of coding potential and division between protein-coding and long non-coding transcripts.

Majority of the lncRNA detection tools use a length cutoff of 200 nucleotides (nt) and sequence intrinsic features and/or statistics to discriminate between coding and non-coding transcripts. The most commonly used sequence features include, for example, open reading frame (ORF) length, Fickett’s score, based on sequence statistics such as position of nucleotides [5] and K-mer composition. A cutoff of 300 nt has been considered as a minimum for ORF length of protein-coding transcripts and a set of di- and trimers has been observed to be more frequent in long non-coding than protein-coding transcripts [6, 7]. However, there are non-coding transcripts with longer ORFs, and vice versa, some mRNAs coding for short peptides have ORFs shorter than this cutoff [8].

Additionally, some tools search for sequence homologies between known protein-coding genes and novel transcripts to aid in categorizing the transcribed RNA sequences as coding or non-coding. However, this is often time consuming, and for many known lncRNAs, no sequence homologies can be found with other non-coding RNAs. Moreover, some lncRNAs have been hypothesized to be derived from protein-coding genes, and share homologies with them (reviewed in [9]). Thus, instead of sequence homology, conserved secondary structures have been proposed to be more frequent [10].

In order to achieve better classification of coding and non-coding RNAs, many different features and their interactions have been added to the list of features differentiating between protein-coding and long non-coding transcripts. However, this inevitably leads to very high-dimensional data. Traditional models, such as logistic regression, can be restricted in the number of features and their interactions that can be included for a well-fitting and well-generalizing model. Machine learning methods such as support vector machines (SVM), are better at handling such data, and thus, they are commonly implemented to train the models based on labelled training data. Nevertheless, these models can still suffer from the increasing computational complexity [11].

In both traditional statistical models and machine learning models, human selected features are introduced as an input and thus the models are often restricted by the current human knowledge. Efforts to circumvent the fallibility of human decision-making have been taken by implementing multi-layered deep learning methods. These methods can handle the given data and/or features without predetermined feature extraction and have the potential to learn hidden structures and dependencies from the data [12]. Thus, using previously unknown information learned from the data, deep learning tools may improve the discrimination between lncRNAs and mRNAs.

In short, deep learning methods combine artificial neural networks with multiple hidden layers for decision-making (reviewed in e.g. [13–15]), allowing linear and non-linear functions to connect the hidden layers. Several different categories of deep learning methods exist based on their preferred architecture, including deep neural networks (DNN), deep belief networks (DBN), convolutional neural networks (CNN) and recurrent neural networks (RNN) [13, 16]. The weight parameters of all hidden layers are optimized and updated with a stochastic gradient decent method [16, 17]. CNNs are built with convolution layers and pooling layers, allowing discovery of new structures by aggregating a complex feature from many small and perhaps similar individual features [13, 16]. RNNs are designed for sequential data, and their cyclic connections enable updating of predicted values. The benefits of using deep learning methods over other machine learning methods in coding potential prediction may arise from handling of multidimensional data and combining known and new features in complex ways for better discrimination. In practice, these possible benefits may manifest as higher sensitivity and specificity when identifying lncRNAs from mRNAs [11].

Coding potential tools have been tested and benchmarked in many setups and datasets [16–19]. However, comparisons have mainly focused on comparing individual tools on a specific test set. To our knowledge, no other independent study has compared the most recent deep learning tools with tools utilizing other modelling methods. In addition, the feasibility of the tools in real-life studies is often bypassed in tool comparisons. Thus, the aim of this study was to test the performance of published tools for lncRNA identification, with the particular goal of comparing tools based on deep learning with other tools. Following the proposed good practice for tool testing [20], we further aimed to test the performance of the tools in a real-life lncRNA-study setup. Furthermore, we evaluated the usability of the tools by scoring the installation and running steps and tested the tools for scalability and robustness with different sized datasets and datasets with biased transcript classes.

## Methods

### Coding potential prediction tools

A subset of 15 tools representing the variety of available methods for coding potential prediction was selected (Table 1). All the tested tools were run with default parameters, species-specific parameters or author recommended parameters unless otherwise stated. The tools were evaluated by their suitability for finding novel lncRNAs, compatibility with the latest reference genome (hg38) and their ease-of-use. The ease-of-use was scored using three-level scoring based on the level of difficulty in the installation and in the running of the tool.

**Table 1:** Long non-coding RNA (lncRNA) identification tools and their features. The usability score was based on ease-of-use in installation and running of the tool. Tools with score of 6 or 7 were selected for performance comparison. Additionally, longdist was included in the comparison, because the problem in running was effortlessly fixed

Tool	Model group (type)	Input format	Trained on/Retraining (yes/no)	Installation	Running	Usability score	Reference
COME	RandomForest	gtf	Hg19 / no	3	2 = only hg19	5	[24]
CPAT	logReg	bed, fasta	RefSeq, GENCODE / Yes	3	4	7	[21]
CPC2	SVM	fasta	Hg19 / yes	3	4	7	[22]
DeepLNC	DeepLearning (NN)	fasta	RefSeq, Lncipedia / yes	NA/3	0	3	[39]
FEElnc	RandomForest	gtf	Hg38, GENCODE 25 / yes	2 = BioPerl installation required;requires much space.	2 = Only ‘.fasta’ accepted for genome file name	4	[40]
IRSOM	NN (SOM)	fasta	Ensemble 92, GENCODE / yes	2 = Featurer needs specific version of compiler	4	6	[25]
iSeeRNA	SVM	gtf, bed	Hg19 / yes (not recommended)	1 = dependent software, preparation of config files	2 = only hg19	3	[41]
lncADeep	DeepLearning (DBN)	fasta	RefSeq 75, GENCODE 24 / yes	3	4	7	[26]
LncFinder	SVM	fasta	GENCODE / yes	3	4	7	[23]
LncRNAnet	DeepLearning (CNN)	fasta	GENCODE 25, Ensembl 87 / yes	3	2 = Does not accept ‘N’ as a nt	5	[42]
lncRScan-SVM	SVM	gtf, fasta	Hg19, GENCODE 19 / yes (not recommended)	3	2 = Many preparation steps, hg19	5	[43]
lncScore	logReg	bed, fasta	GENCODE 23 / yes	3	0 = Error: ‘linear model not converging’	3	[44]
longdist	SVM	fasta	Hg19, GENCODE 19 / yes (not recommended)	3	2 = Error in attribute list format needed to be fixed	5	[7]
mRNN	DeepLearning (RNN)	fasta	GENCODE 25 / yes	3	4	7	[11]
RNAsamba	DeepLearning (RNN)	fasta	CPC2, FEElnc, mRNN / yes	3	4	7	[27]

Tools and related packages and software, if applicable, were downloaded following the given instructions by the developers. We did not train any of the models, in order not to give advantage to those tools that could be trained, over those that cannot be trained. Of those tools that provided both lncRNA prediction and lncRNA function prediction, we only used the lncRNA prediction. The full commands for each tested tool can be found in Supplementary File 1.

The rating of the tools for ease-of-use (usability score) was calculated by summing the installation (0–3) and running (0–4) scores together, giving higher weight for good running performance, since most of the user experience is related to running the tools. Tools with an overall score of 6 or 7 were included in the more detailed performance evaluation. Additionally, the tool longdist was included, although it scored 5 due to erroneous formatting of the feature list, since the solution to the problem was provided on the tool’s homepage and was easily fixed. The eight tools selected represented most model types and included the following tools: three deep learning tools (LncADeep, mRNN and RNAsamba), one self-organized neural network (IRSOM), three SVMs (CPC2, LncFinder and longdist) and one logistic regression model (CPAT). Here we present the tested tools briefly under the corresponding model types.

#### Logistic regression

**CPAT** [21] is a much-cited alignment-free tool for coding potential prediction that is based on a logistic regression model of four variables: maximum ORF length, relative ORF length, Fickett Score of nucleotide composition and codon usage bias, and hexamer score for hexamer usage bias between lncRNAs and mRNAs [21]. The logistic regression model, using these features as explanatory variables, was built and fitted to training data of selected protein-coding sequences and random lncRNA sequences. A binary decision-making algorithm was then constructed based on the calculated features. The CPAT version 2.0 comes as a python 3 installation through pip and it can be re-trained. In this study, we used the Human_logitModel and human_hex.tab provided with the tool from the pre-trained model as the input in the tool testing (Supplementary File 1).

#### Support vector machine

**CPC2** [22] is a popular trained SVM on four features. Similarly, as CPAT, CPC2 uses Fickett Score and ORF length as classifying features. In addition, the CPC2 features include ORF integrity, for checking if the ORF is complete with start and stop codons, and the theoretical isoelectric point of the forming peptide. As an upgrade to the older version of CPC, CPC2 is not dependent on alignment-based methods. CPC2 version 0.9 was used, and the command line version is implemented through bash and perl commands.

**LncFinder** is an SVM model predicting lncRNAs and mRNAs using nine feature extraction functions packaged in R [23]. LncFinder uses sequence-based features, secondary structures and physicochemical properties. The sequence-based features include length and coverage of the longest ORF, and the logarithm-distance of hexamer on ORF. The structural features measure the minimum free energy of the secondary structure, indicating the structure’s stability, and depict three secondary structure features: frequency of paired and unpaired sequences and logarithm-distances of acguD and acgu–ACGU-sequence. In addition, LncFinder uses three features calculated from the electron-ion interaction potential to capture the energy of each nucleotide and the signal of the sequence. LncFinder version 1.1.3 was used in this study and it requires R version 2.10 or higher. LncFinder was run without secondary structure information, setting SS.features = FALSE in the lnc_finder function, as was recommended for feasible running times if secondary structure was not readily available. Frequencies file for human was provided with the tool.

**Longdist** is an SVM model trained using nucleotide composition and ORF-features [7]. The model has been built using principal component analysis (PCA) to select 50 nucleotide di- and trimers best capturing the differences in pattern frequencies between lncRNAs and mRNAs. In addition, the length of the first ORF of a transcript was found to capture the differences well and was thus included as a feature in the model. Longdist version 1.0.3 was used in this study, which was trained on both hg19 and hg38. The tool runs through a python 3 implementation and can be easily installed using pip. However, at the time of the testing, before running the tool, an error in the configuration file must be manually fixed with the solution from ‘https://github.com/hugowschneider/longdist.py’. After the code fix, the tool was run with the provided GRCh38_firstOrf-model.

#### Self-organized neural network

**IRSOM** applies a three-layer neural network to lncRNA coding potential prediction, including input layer, self-organizing map (SOM) and output layer [25]. Transcript feature vectors, composed of sequence and ORF-based features, are compared to neuron unit clusters in the SOM. The network is then updated by a function consisting of the learning rate, network neighborhood function, and the Manhattan distance between the current neuron unit and other clusters. The sequence features include k-mer motif frequencies, codon position bias, nucleotide frequencies and GC content. The ORF-based features include the coverage of the longest ORF, ORF coverage distribution, start and end codon distribution, ORF frequency, ORF length and frame bias. Unlike many other tools, IRSOM provides the opportunity to reject unreliable predictions if the labelling is uncertain. IRSOM implementation was run on python 3.

#### Deep learning

**LncADeep** is a deep learning-based tool designed to both predict the coding potential and the function of novel transcripts [26]. Here we tested the coding potential capabilities of the tool. LncADeep integrates sequence intrinsic features and homology features into a DBN, with three restricted Bolzmann machines stacked between the input and output layers. By default, LncADeep can identify both full- and partial-length transcripts, missing either 3′ or 5′ end untranslated regions (UTRs), or even uncomplete coding sequences. The sequence-based features included in LncADeep are ORF length and coverage, entropy density profiles (EDP) based on amino acid frequency, k-mer composition (16 dimers), mean hexamer score, UTR coverage, GC content and Fickett score of nucleotide composition. The homology-based features consist of sequence conservation scores from HMMER search against Pfam (Release 29.0), including alignment score, and the ratio of the aligned region to the query sequence. Majority voting is used to decide the final labelling of a transcript [26]. LncADeep version 1.0 was used, implemented through python 2.7, R v3.3.2 and HMMER (3.1b2). In this study, LncADeep was run with -MODE lncrna for lncRNA identification. Parameter—model was kept default for the model for partial-length transcripts.

**mRNN** applies a Recursive Neural Network (RNN) directly on input RNA sequence data using one-hot encoding for the sequencing [11]. Gated recurrent unit architecture was used to manage memory and improve learning of long-range dependencies in the hidden layers. The post training experiments revealed that mRNN learned to distinguish mRNAs from other transcripts by trimer patterns, lack of in-frame stop codons in an ORF, and a set of 11 enriched codons, significantly represented in protein-coding transcripts. In addition, important connections between distant codons were revealed by a point mutation analysis. Implementation of mRNN uses python 2.7 with Theano library and a specified version of Passage (https://github.com/IndicoDataSolutions/Passage). For the tool testing, we used the pre-calculated weights from the model file w16u5-plk.

**RNAsamba** implements a neural network model using IGLOO architecture for its CNN, designed for long sequences [27]. The input sequence is handled in two branches, one of which uses the whole sequence, whereas the other processes ORF information from the longest ORF, only if a start codon is found. The only predefined information given to RNAsamba is the start codons in order to identify ORF. The RNAsamba model has been trained using both complete and truncated sequences. RNAsamba is implemented using python 3 with TensorFlow and Keras libraries and can also be run online at https://rnasamba.lge.ibi.unicamp.br. In this study, we tested RNAsamba using the weights from the pre-trained partial-length model.

### Benchmarking with reference test set

We used protein-coding transcripts from GENCODE (GRCh38) and long non-coding transcripts from LNCipedia, version 5.2 [28] for the tool testing. Only level 2 validated protein-coding transcripts, and high confidence lncRNA transcripts were included to minimize errors in the test data. In order to alleviate possible differences arising from the different training sets used in the original studies (Table 1), we selected the overlapping protein-coding transcripts between GRCh37/hg19 and GRCh38/hg38. Further, the long non-coding transcripts present in both GENCODE and LNCipedia, were removed from the lncRNA transcripts. Transcript positions were taken from the provided LNCipedia reference files in GTF-format, and transcript sequences were extracted from the reference genome (GRCh38) with gffread [29].

The different coding potential prediction tools were tested with four different sized test sets. The test sets were generated by first randomly dividing all long non-coding transcripts from LNCipedia and all protein-coding transcripts from GENCODE to five subsets. Four subsets from both types of transcripts were then used and combined to make four test sets with the smallest test set (named S, subset Lnc-A + subset Pc-A) containing 46 563 transcripts, the test set M (subsets Lnc-A + Lnc-B + subsets Pc-A + Pc-B) containing 92 922 transcripts, the test set L (subsets Lnc-A + Lnc-B + Lnc-C + subsets Pc-A + Pc-B + Pc-C) containing 139 379 transcripts, and the biggest test set XL (subsets Lnc-A + Lnc-B + Lnc-C + Lnc-D + subsets Pc-A + Pc-B + Pc-C + Pc-D) containing 185 190 transcripts (Table 2). Due to differences in transcript numbers between protein-coding and lncRNA references, each test set contained 41% lncRNAs and 59% mRNAs. Further, in order to test the robustness of the tools towards biased datasets, two imbalanced test sets were considered: a test set containing 78% lncRNA transcripts (named Lnc_bias test set, subsets Lnc-A + Lnc-B + Lnc-C + Lnc-D + subset Pc-A) and another test set containing 85% protein-coding transcripts (named Pc_bias, subset Lnc-A + subsets Pc-A + Pc-B + Pc-C + Pc-D) (Table 2).

**Table 2:** Reference test sets of annotated transcripts. The total number of transcripts, the number of protein-coding (mRNA) and long non-coding (LncRNA) transcripts, and the proportion of the long non-coding transcripts are shown

Test set	Total transcripts	LncRNA	mRNA	Proportion LncRNA
S	46 563	19 069	27 494	40.95%
M	92 922	38 008	54 914	40.90%
L	139 379	56 967	82 412	40.87%
XL	185 190	75 757	109 433	40.91%
Lnc_bias	122 265	94 771	27 494	77.51%
Pc_bias	128 502	19 069	109 433	14.84%

Sequence fasta files of each test set were given to the tools as input. Tools were run in a local computer cluster running linux (Centos 7.7) with Intel Xeon Gold processors. Each tool was run in a job looping over all test sets with 55.2 GB memory on 12 processor units (Supplementary material). As an exception, LncFinder needed to be run by specifying 64 GB memory for a successful use of the tool. Total running time was measured for each tool for each test set.

Tool outputs were parsed and figures drawn with custom python scripts. A threshold of 0.5 was used as a probability cutoff for binary labels, if a threshold was not given by the tool or the tool did not readily label the transcripts as coding or non-coding. All tested tools considered coding transcripts as positive and long non-coding transcripts as negative sets.

Tool performance on each test set was evaluated by determining the confusion matrix (Table 3). Sensitivity, specificity and precision were then calculated from the numbers of true positive (TP), true negative (TN), false positive (FP) and false negative (FN) labels (Table 3). Further, both receiver operating characteristic (ROC) curve and precision-recall curve (PRC) were drawn and areas under the curves for each test set were calculated to compare tool performances. ROC curves best capture the performance on the balanced test sets, whereas PRCs are suitable measures for unbalanced test sets particularly when the optimal threshold is not always known [30, 31]. Measures were calculated and figures drawn with python package scikit-learn (version 0.22.2.post1).

**Table 3:** Confusion matrix and the calculated metrics in evaluating tool performance. In Jaccard similarity index, tool specific labelling for given test set is treated as sets A or B for the compared tools respectively. Set comparisons are thus based on the similarity of the labels given to each transcript

Performance evaluation metric	Formula/layout	
Confusion matrix	TP, true positives, hits	TN, true negatives, correct rejection
	FP, false positives, type I error	FN, false negatives, type II error
Sensitivity, true positive rate, recall	$\frac{TP}{TP+ FN}$	
Specificity, true negative rate	$\frac{TN}{TN+ FP}$	
Precision	$\frac{TP}{TP+ FP}$	
Jaccard similarity index J (A,B) Jaccard distance = 1−J (A,B)	$\frac{\Big|A\cap \Big.\Big.B\Big|}{\Big|A\cup \Big.\Big.B\Big|}$	

### Testing tools on real-life data

As real-life datasets, we used RNA-seq data on human cell types associated with the innate immune responses also analysed in a published study [32]. These datasets were chosen due to recommended read length for novel transcriptome assembly and sufficient read depth for discovering lncRNAs that are potentially very lowly expressed. The datasets included paired-end RNA sequence reads of length 100 bp from stimulated and non-stimulated human monocyte, macrophage, epithelium and chondrocyte samples. Reads were downloaded from the following entries in Gene Expression Omnibus: GSE101868, GSE74220 and ERA294222. Altogether there were 27 samples with an average of 38.9 million reads each.

The reads were aligned with STAR-2.6.1b [33] two pass mode, guided by the UCSC reference (hg38) junctions. Individual samples were then run through Stringtie version 2.0.6 [34] and all samples were collected into a master transcriptome with Stringti – merge. A minimum length of 200 nt and fragments per kilobase million (FPKM) of at least one were required for the included transcripts. Other Stringtie parameters were kept as default. Gffcompare [29] was used to separate transcripts matched to reference (UCSC hg38) and novel transcripts. Similarly to the original study [32], multi-exonic novel intergenic lncRNA (lincRNA) and antisense RNA loci were included in the tool testing. The sequences of the identified novel transcripts from all cell types were saved as a combined fasta file using Gffread [29] and used as input for the different tools. Tools were run on the real-life datasets in the same computer cluster with the same specification as the reference test sets.

Tool outputs were parsed and analysed with custom python scripts. As a complete true set is unavailable for the real-life dataset, regular measures for tool performance, such as sensitivity and specificity, cannot be calculated. Instead, the proportion of lncRNA and mRNA labels were recorded from each tool and compared. In addition, the similarity of the tools’ categorization was measured by calculating Jaccard index of similarity for the predicted labels (lncRNA/mRNA). The similarity was calculated using set operations on transcript names that were tagged with tool-given labels. In order to see, which tools were more similar with each other, Jaccard distance matrix, calculated from 1-Jaccard similarity, was clustered by hierarchical clustering using average linkage.

## Results

**Figure 1:**
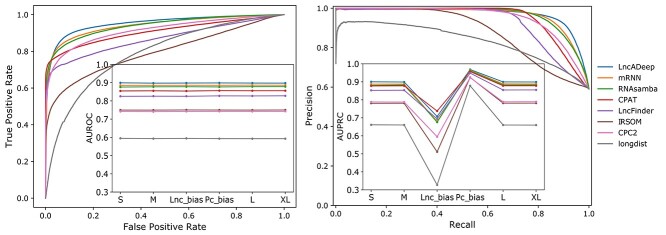
Performance of the coding potential prediction tools in the reference test sets for all tested tools. (A) An example Receiver Operator Characteristics (ROC) curve of the test set M and the area under the ROC curve (AUROC) for all test sets (S, M, L, XL, Lnc_bias and Pc_bias; inset). (b) Precision-recall curve (PRC) and area under PRC for all test sets (S, M, L, XL, Lnc_bias, Pc_bias; inset). The test sets (*x*-axis) were ordered on the basis of the total number of transcripts in the test set. ROC and PRC figures for other test sets can be found from Supplementary Figures 1 and 2.

We first tested the ability of the eight tools to separate lncRNAs from protein-coding transcripts using six reference test tests. In all of the test sets, the performance and the order of the different tools remained similar when measured with the area under ROC curve (AUROC, Figure 1a, Supplementary Figure 1). The three deep learning tools (LncADeep, mRNN and RNAsamba) were at the top with mean AUROC values of 0.90 (±0.0005 standard error of the mean, SE), 0.89 (±0.0003 SE) and 0.88 (±0.0007 SE), respectively. Among the other tools, CPAT had the highest mean AUROC value of 0.86 (±0.0006 SE), while had the lowest AUROC values throughout the datasets, with a mean of 0.59 (±0.0004 SE).

The PRC was used to capture the tool performance especially on the unequal test sets containing a large proportion of lncRNA or protein-coding transcripts (Lnc_bias and Pc_bias, respectively). As expected, PRC was affected by the proportion of lncRNA and mRNA transcripts in the test sets (Figure 1b, Supplementary Figure 2). In general, the tool performance order stayed the same as when measured with AUROC, with LncADeep leading, followed by mRNN and RNAsamba. The only exception was the biased test set towards lncRNAs (Lnc_bias), where CPAT performed better than and LncFinder performed more similarly to the deep learning-based methods. All the tools performed well on Pc_bias test set, which could be expected based on the general trend of training sets being more mRNA biased and potentially including the reference mRNAs used in testing.

**Figure 2:**
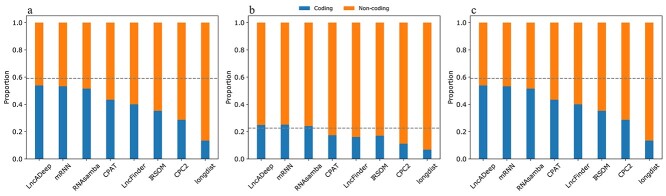
Proportions of predicted non-coding or protein-coding transcripts by the tested tools. Proportions for (A) reference test set M, (B) reference test set with a large proportion of non-coding transcripts Lnc_bias and (C) reference test set with a large proportion of protein-coding transcripts Pc_bias are shown for comparison. The proportion of predicted non-coding and protein-coding transcripts is marked in orange and blue, respectively. The true proportions for each test set are indicated with horizontal dashed lines. Figures of predicted proportions for other test sets can be found in the Supplementary Figure 3.

The ability of the tools to respond to the different proportions of lncRNAs and mRNAs was further visualized by comparing the predicted versus actual proportions of these two types of transcripts (Figure 2, Supplementary Figure 3). The proportions of transcripts labelled as lncRNAs or mRNAs by the deep learning tools were closest to the real proportions of these transcripts in each reference test set (Figure 2A) and they followed more closely the changing proportions of the classes in the biased test sets compared to the rest of the tools (Figure 2B and C). In particular, longdist seemed to predict high numbers of lncRNA transcripts irrespective of the actual proportion of these transcripts in the test set (Figure 2).

**Figure 3:**
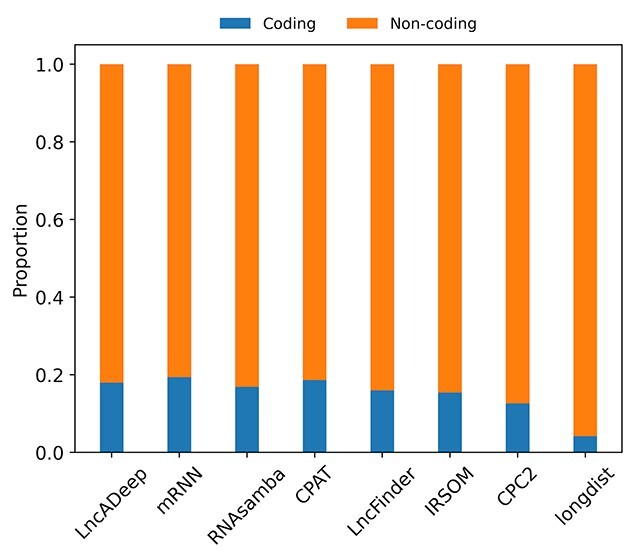
Proportions of predicted non-coding and protein-coding transcripts in the real-life dataset. The proportion of predicted non-coding and protein-coding transcripts for each tested tool is marked in orange and blue, respectively.

We further tested the performance of the tools on the previously published real-life data on human monocytes, macrophages, chondrocytes and epithelial cells [32]. In total, 3219 novel loci were identified from the data across cell types. All tools labelled over 80% of them as lncRNAs (Figure 3). The order of the deep learning tools in the proportion of labelled loci was the same for the real-life data as for the Lnc_bias reference test set. Again, longdist predicted the largest proportions of lncRNA transcripts, labelling 96% of the transcripts as lncRNAs. Although CPAT labelled slightly higher proportions of transcripts as lncRNAs than the deep learning tools in the reference test sets, in the real-life data the proportions were similar. As the deep learning tools followed best the known proportions of protein-coding and non-coding transcripts in all the reference test sets, we may anticipate that the real proportion of lncRNA loci in these data is ~80%.

**Figure 4:**
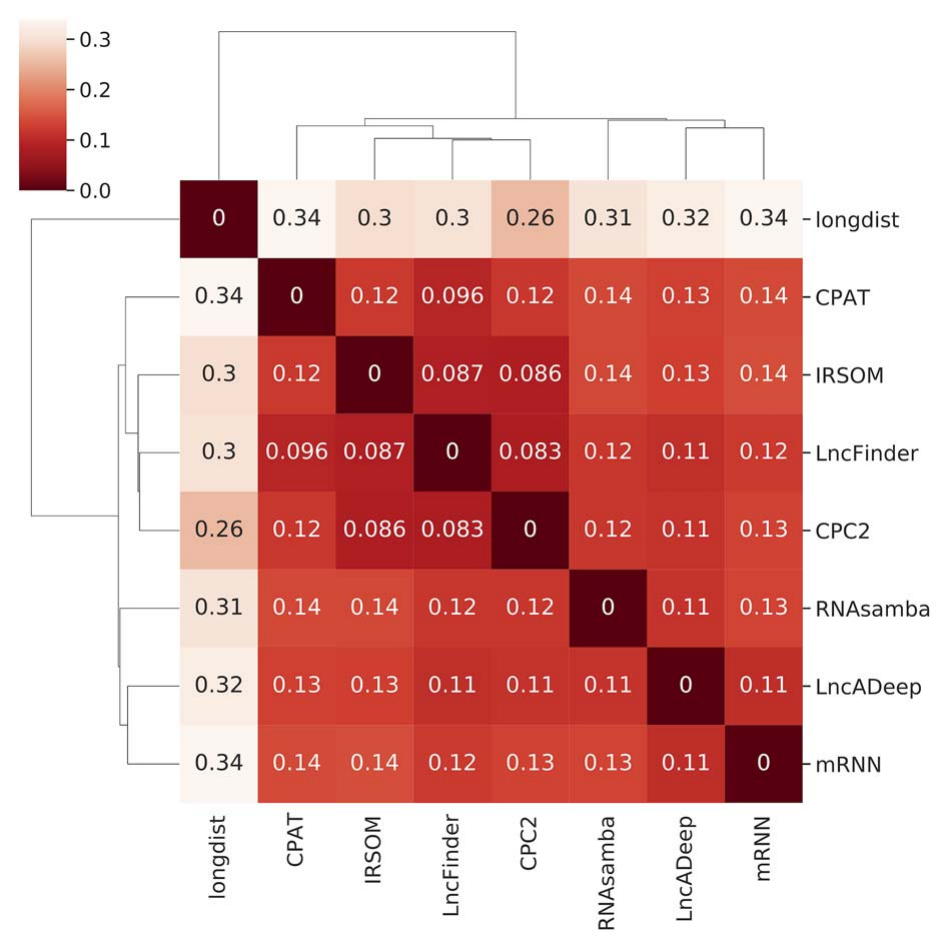
Hierarchical clustering of tools, based on Jaccard distance of tool-given labels for the real-life data. Jaccard distance calculations were performed both ways, resulting in identical values and clustering.

To further investigate the similarities and differences between the tools in the real-life data, we assessed the agreement of the predictions between the tools across all the novel transcripts found in this study. Overall, the tools labelled between 66% (mRNN versus longdist) to 92% (CPC2 versus LncFinder) of the transcripts concordantly as lncRNA or mRNA. Hierarchical clustering of the Jaccard distance of the tools revealed three main clusters (Figure 4). Longdist labelled the transcripts most distinctly compared to all the other tools, which is seen as its own branch in the hierarchical clustering. Another branch was formed by IRSOM, CPC2, LncFinder and CPAT, with ~90% agreement of the labels when compared to each other. In the third branch, the three deep learning tools, RNAsamba, LncADeep and mRNN clustered together labelling ~90% of the transcripts in agreement with each other.

**Figure 5:**
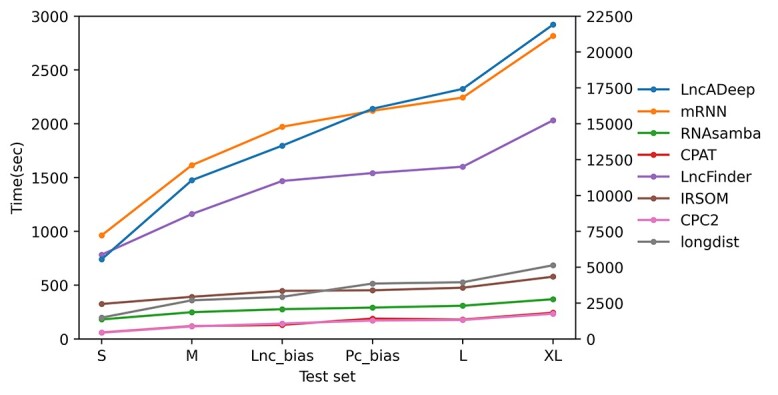
Total run times (seconds) of the tested tools under the same conditions, for all the reference test sets (S, M, L, XL, Lnc_bias, Pc_bias). The test sets (*x*-axis) were ordered on the basis of the total number of transcripts. Note that the values for LncADeep are plotted on the secondary *y*-axis, whereas the rest of the tools are plotted on the primary *y*-axis.

In addition to performance, another important question when choosing a tool is the run time. There were apparent differences in the run times between the tools (Figure 5). CPC2 and CPAT were the fastest tools, with run times from 58 s (test set S) to 243 s (test set XL). The next fastest tool was the deep learning tool RNAsamba with run times ranging from 181 s to 369 s, being clearly to fastest among the deep learning tools. The other deep learning tools LncADeep and mRNN were the slowest of all the tools, with run times ranging from 16 min up to 6 h, depending on the size of the data. The run time of some of the tools seem to have been affected by the biased test classes, whereas others were likely only affected by the different numbers of transcripts (Figure 5).

## Discussion

The identification of lncRNAs suffers from many stages that introduce uncertainty to the results. One of them is the estimation of the coding potential of the novel transcript, often from incomplete transcripts. Deep learning methods have been increasingly utilized in addressing the potential sources of error. Here we compared a set of previously introduced tools designed to assess the coding potential of a given transcript to separate lncRNAs from mRNAs. In particular, we concentrated on the potential differences between the tools utilizing deep learning methods and other machine learning approaches. As evaluation criteria, we used performance in labelling the transcripts correctly, capability of the tools to perform on uneven datasets, and usability of the tool as a whole.

No tool was clearly best in all performance test metrics and evaluation criteria. However, the three tested deep learning tools (LncADeep, mRNN and RNAsamba) were always among the top performing tools, followed by the logistic regression based CPAT and the SVM based LncFinder. In particular, the deep learning tools distinguished best between lncRNAs and mRNAs in the reference test sets. The deep learning methods finished at the top also, when performance was measured in the proportions of mRNAs and lncRNAs labelled by the tools, compared to the actual proportions (Figure 2). The proficiency of the deep learning tools at agreeing with the true labels indicates that the correct proportions were not achieved due to incorrect labels in right proportions. CPAT and LncFinder also followed the true proportions well, though tending to slightly overestimate the number of lncRNA transcripts in the datasets.

The biased test sets, Lnc_bias and Pc_bias, caused all tools to fluctuate in their ability to correctly label the transcripts to the same direction. Since the PRCs do not consider both classes equally, we applied them both ways for the biased test sets (Supplementary Figure 4). This suggested that the deep learning tools indeed rank at the top at predicting lncRNAs.

LncADeep was the highest ranking deep learning tool measured as AUROC and AUPRC. It uses a DBN to learn the distinction between lncRNAs and mRNAs based on sequence and homology features. Other tools, for example LncFinder, implement similar and/or the same features, with less success in correctly labelling transcripts. Therefore, it might be that either the number of included features is responsible for the success of LncADeep, or that the network architecture is capable of learning new associations between the features, thus improving the success in labelling.

Unlike LncADeep, the deep learning architectures implemented by mRNN and RNAsamba utilize directly the sequence data and discern lncRNAs and mRNAs based only on the sequence and any structures/features that can be learned from it. These deep learning architectures outperformed many other tools with fixed input feature vectors. Thus, they likely learned features or feature interactions beyond the current human knowledge about the differences between lncRNAs and mRNAs.

The huge potential of deep learning tools to recognize previously unknown patterns from sequence data comes with the difficulty of interpreting these patterns afterwards. The interpretation of the patterns that deep learning tools have used to discern between coding and non-coding transcripts relies on separate interpretability analyses. Based on the analyses of the developers of mRNN and RNAsamba, the tools were able to utilize known sequence features in their decision-making [11, 27]. Further, mRNN used previously unknown sequence information named translation indicating codons (TICs) in defining the sequence coding potential [11].

The tools’ performance on the real-life dataset was quite similar to that on the reference data sets (Figure 3). The deep learning tools and CPAT labelled similar proportions of transcripts as lncRNAs to each other, whereas longdist in particular tended to overestimate the proportion lncRNAs. Hierarchical clustering with Jaccard distance placed the three deep learning tools in the same branch, with distances of around 12% between each other. This indicates that the deep learning tools labelled almost 90% of the transcripts in the same way. Known reference test set transcripts were labelled by CPAT and LncFinder with a small difference in AUPRC compared to the deep learning tools. The labels by CPAT and LncFinder for the real-life dataset were between 11% and 14% different to those of the deep learning tools. On the other hand, CPAT and LncFinder were more similar to each other, with only 9.6% difference. These differences may indicate that the top tools make slightly different choices or mistakes in labelling novel transcripts. All the deep learning tools have considered incomplete transcripts in their design. Since real-life data processing of RNAseq data often results in many incomplete transcripts, the differences in labelling may arise from this particular type of transcripts.

Tools with similar features and implementation did not guarantee similar results in transcript labelling. IRSOM (SOM), CPC2 (SVM) and LncFinder (SVM) were most similar with each other in the real-life dataset and the performance of IRSOM and CPC2 were alike on the reference test sets. These tools all use similar ORF-features but differ in the use of secondary structure features; CPC2 and LncFinder use secondary structure, whereas IRSOM does not. On the other hand, longdist also uses very similar sequence features to IRSOM (k-mers and ORF-based features) and is implemented as an SVM, like CPC2 and LncFinder. Nevertheless, longdist was the most dissimilar of all the tools and did not perform as well on the reference test sets. IRSOM may have picked up some relevant sequence characteristics better from the SOM-network than longdist with SVM. Thus, not only the features and the implementation but perhaps the combination of the two affected the performance of the tools utilizing other than deep learning methods.

Parameter choices and test sets affect tool performance a great deal. In this study, LncADeep was run with default parameters and performed best in terms of correctly labelling the reference test set transcripts. However, LncADeep did not perform well in all previously published comparisons [16, 19 but see 33]. According to Yang *et al.* [36], LncADeep may have been run with suboptimal parameters in the previous comparison with lncRNAnet and lncFinder [16 but see 35]. Although Xu *et al.* [19] ran LncADeep with recommended parameters, the python 2 code was modified to be compatible with python 3, with unknown effects on performance. In addition, these studies tested the performance on hg19 Havana annotated reference set [16] and a selected small subset of NCBI Ref Seq annotated sequences [19] while in comparison, this study used non-coding transcripts from LNCipedia specifically excluding lncRNAs present in GENCODE, which many tools use in training. Few of the tested tools allow for parameter changes outside selecting the study organism and between pre-trained model weights to use in the models. Lack of parameter choices makes the tool user friendly in its straightforwardness, however a real-life project could benefit from parameter optimization and/or retraining the models where applicable.

Real-life data run with lncRNA prediction tools differ greatly from the reference transcripts used in model training. Most pipelines for lncRNA identification filter out known lncRNAs and mRNAs before aiming to identify new lncRNAs from among the novel transcripts. Thus, when lncRNA prediction tools are applied on real-life data, the data may exclude many full-length transcripts and is almost always going to be biased towards lncRNAs. It is good practice for any machine learning method to be trained on balanced dataset in order not to include a bias in the model. However, as could be seen from the results of this study, the performance of the tools on biased datasets may be affected. The overall performance of the deep learning tools remained high despite biased datasets, however, future efforts could take the biased nature of the set of transcripts from experiments more into account [15].

Based on our ranking of the overall user experience and the results of the reference and real-life test sets, deep learning tools are a good choice for the task of discerning lncRNAs from mRNAs. The ease-of-use score and user experience overall depend on the user’s experience with command line and/or specific coding language tools. Of the best performing tools only RNAsamba and CPAT can be run as online versions. Thus, they can be run without using local computational resources or much knowhow of different interpreters and be suitable for many kinds of projects. However, so far, the online version of CPAT supports only the older version of the human genomic reference (hg19) for bed-input files. RNAsamba is the only deep learning-based tool that can be run online and it is not dependent on the reference genome. RNAsamba also performed slightly better on the test sets than CPAT. Therefore, the installation free online version of RNAsamba may be the best choice for projects with less computational resources and expertise.

The trade-off for selecting the overall highest scoring tool measured in AUROC and AUPRC, LncADeep, is the computing time. Overall, the time cost increased with the increasing number of tested transcripts for all tools irrespective of the initial training set sizes used by each tool developer. However, the time cost of LncADeep was clearly higher, likely due to calculating alignment-based features. This might weigh heavily in the tool selection decision, especially if computing time is limited and/or costly. If computing time is the most limiting factor to a project, RNAsamba might be the best choice, balancing good performance, easy installation through python pip or online based usage and fast running time compared to the other deep learning tools.

LncFinder was the only tested tool that was written and run through R. R packages are easy to install, and many bioinformatics workflows are based on R programs. The performance of LncFinder was quite good, although several other tools surpassed it in various performance metrics. Further, LncFinder required significantly more computational memory during testing (64 Gt, Supplementary material), and may thus not be an option for some projects.

This study compared a representative set of tools implementing deep learning methods to a representative selection of other methods. Thus, not all existing tools were tested and compared against deep learning tools. Other deep learning tools, for example lncRNA_MDeep [38] and DeepCPP [35], are also available, covering variations of the architectures tested here (DNN/DBN, CNN and RNN). The inhouse benchmarking by Zhang *et al.* [35] included LncADeep, mRNN and RNAsamba, concluding that LncADeep performs well on regular human test set. In addition, the testing supports conclusions from this study, that deep learning tools outperform tools based on other model types.

Many of the initially tried and hence published tools seem not to have been maintained after their publishing, or some parts of their code have become depreciated. This is an unfortunate trend, since many good tools have been developed. Xu *et al.* [19] aimed to overcome this problem by developing a unified python wrapper for a selection of available tools. Furthermore, web interfaces independent of user systems, such as for RNASamba and CPAT help in adopting the best tools for wider systematic use in lncRNA prediction. We chose not to re-train any of the tested tools in order not to give advantage over tools that cannot be re-trained. It is likely that some tools would have performed better if trained on the current data, especially if the training was done on a previous version of the human genome. However, in order for a machine learning method to be broadly used, the model should be general and work on other datasets as well. Thus, the results here may also reflect the generality of each tool.

To conclude, the results of this study showed that deep learning tools LncADeep, mRNN and RNAsamba outperformed most of the tools using other machine learning methods in nearly all datasets tested. The choice of the tool in a real-life project is not straightforward, as the computational and time resources may have to be weighted in order to choose the best deep learning tool for the task. In many studies, accuracy may weigh heavier in the choice of the tool than computational resources leaning the decision towards LncADeep. In other studies, mRNN or the online version of RNAsamba may be preferred. Irrespective of the choice, with the potential of taking into account features or feature interactions not included in current human knowledge, deep learning tools offer a fast and reliable choice for lncRNA identification, beyond what machine learning methods with only predetermined features can offer at the moment.

**Key Points**

- The difficulty of identifying long non-coding transcripts from sequence data has been approached by tools using statistical and machine learning methods, and lately, deep learning methods.
- Deep learning tools LncADeep, mRNN and RNAsamba ranked at the top in predicting the coding potential measured in terms of prediction performance and in ease-of-use when compared to other methods.
- Additional benefits from using deep learning methods may be brought upon from utilizing features not incorporated in the current human knowledge.

## Data availability

Sequence data used in benchmarking the tools are available through GENCODE and LNCipedia pages. Real-life data can be accessed through Gene Expression Omnibus with the accession numbers GSE101868, GSE74220 and ERA294222. Each tested tool was available on their respective home pages in May 2021. Code used for running the tools can be found in the Supplementary material. Any other code can be accessed through a request to the corresponding author.

## Supplementary Material

Ammunet_etal_Supplementary_toolComparison_revised_final_elab045

## Notes

**Tea Ammunét** is a post-doctoral researcher at the Medical Bioinformatics Centre at the University of Turku, Finland. Her expertise lies in long non-coding RNAs and statistical methods in bioinformatics.

**Ning Wang** is a doctoral student in bioinformatics at the University of Turku supervised by Professor Laura Elo.

**Sofia Khan** is a post-doctoral researcher at the Medical Bioinformatics Centre at the University of Turku, Finland. Her research interests include human genomics and computational sequence analysis.

**Laura L. Elo** is Professor of Computational Medicine and Head of Medical Bioinformatics Centre, University of Turku, Finland. Her main research interests include computational biomedicine and bioinformatics.
