# Supplementary material for: Deep learning tools are top performers in long non-coding RNA prediction
Source: Brief Funct Genomics. 2022 Feb 6;21(3):230–41. doi: 10.1093/bfgp/elab045 (PMC9123429; doi:10.1093/bfgp/elab045)
Supplement: Ammunet_etal_Supplementary_toolComparison_revised_final_elab045 [file ammunet_etal_supplementary_toolcomparison_revised_final_elab045.zip › Ammunet_etal_Supplementary_toolComparison_revised_final_elab045.docx]

**Supplementary material for Ammunet et al. “Deep learning tools are top performers in long non-coding RNA prediction”**

**Running codes for tool testing**

Test sets were located in a directory named test_sets inside the directory with all tool folders. All tools were tested in the following or similar loop, changes taking place in the file location path depending on the running location requirements for each tool:

outf=”path for output”

For file in ./test_sets/*fa;do

“code for tool run”

done

Below the detailed running codes and parameters are listed for each tool.

**CPAT**

cpat.py -g "$file" -d ../CPAT/Human_logitModel.RData -x ../CPAT/human_hex.tab -o ../CPAT/"$outf"

**CPC2**

./bin/run_predict.sh “full_path_to”/"$file" ../$”outf" . evidence;

**IRSOM**

python3 "${path_to_IRSOM_dir}"/scripts/predict.py --featurer= ”$path_to_IRSOM_dir”/bin/Featurer --file="$file" --model="$outf" --output="$ outf"

**LncADeep**

python "$path_to_lncaDeep_dir"/LncADeep.py -MODE lncRNA -f "$file" -o "$outf" -th 12

**LncFinder** (R code, memory requirements set to 64G)

files<-list.files(test_sets,pattern=".fa")

for (i in 1:length(files)){

tic(files[i])

seqs<-seqinr::read.fasta(file=paste0(path,"/",files[i]))

pred_results<-lnc_finder(Sequences = seqs, SS.features=FALSE,format="DNA",frequencies.file="human", svm.model="human",parallel.cores=-1)

toc()

write.csv(pred_results, paste0(files[i],"_result.csv"))

}

**longdist**

python "$path_to_longdist"/longdist.py --predict --input "$file" --model_config "$path_to_longdist"/models/GRCh38_firstOrf.plk.conf –out "$path_to_longdist"/"$outf".csv

**mRNN**

python "$path_to_mRNN"/mRNN.py -w "$path_to_mRNN"/weights/w16u5.pkl -o "$path_to_mRNN"/outf" -f "$path_to_mRNN"/"$outf" "$file"

**RNAsamba**

rnasamba classify "$path_to_RNAsamba"/"$outf".csv "$file" "$path_to_RNAsamba"/partial_length_weights.hdf5

**ROC figures for all reference test sets**


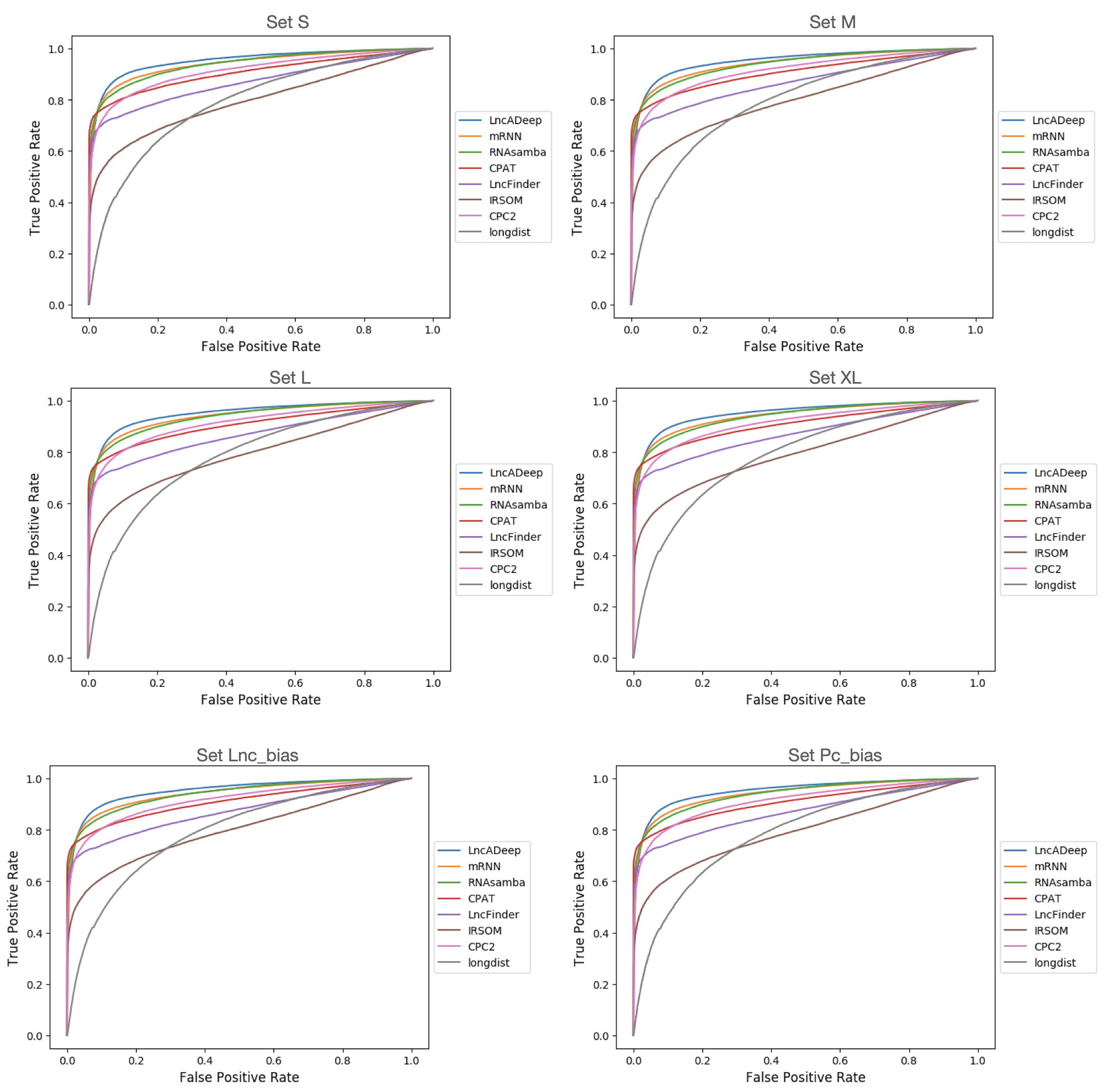


Supplementary Figure 1: Receiver Operator Characteristics (ROC) curve for all reference test sets S, M, L, XL, Lnc_bias and Pc_bias.

**PRC figures for all reference test sets**


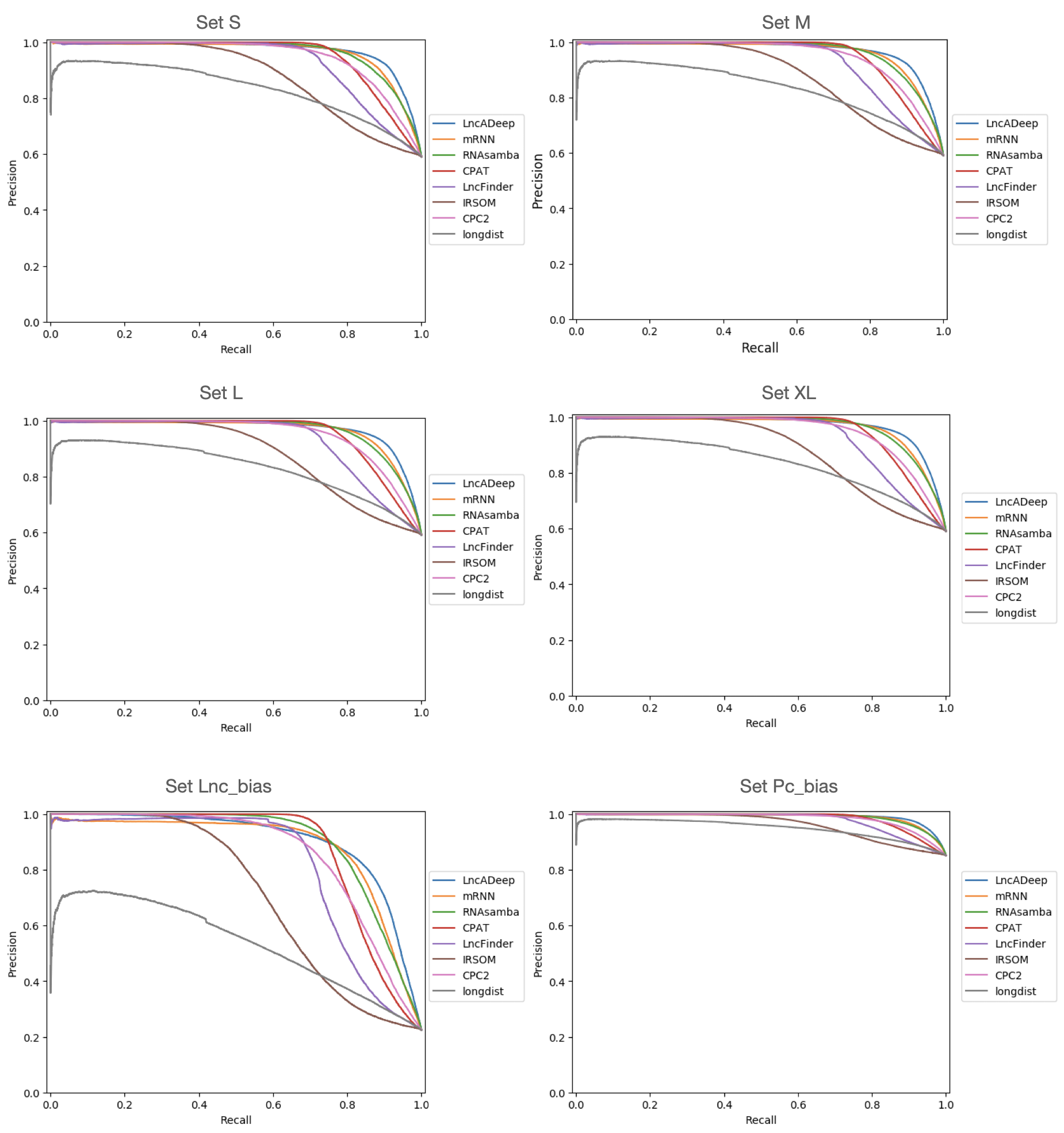


Supplementary Figure 2: Precision-recall curve (PRC) for all test sets (S, M, L, XL, Lnc_bias, Pc_bias).

Proportions of predicted classes for all reference test sets


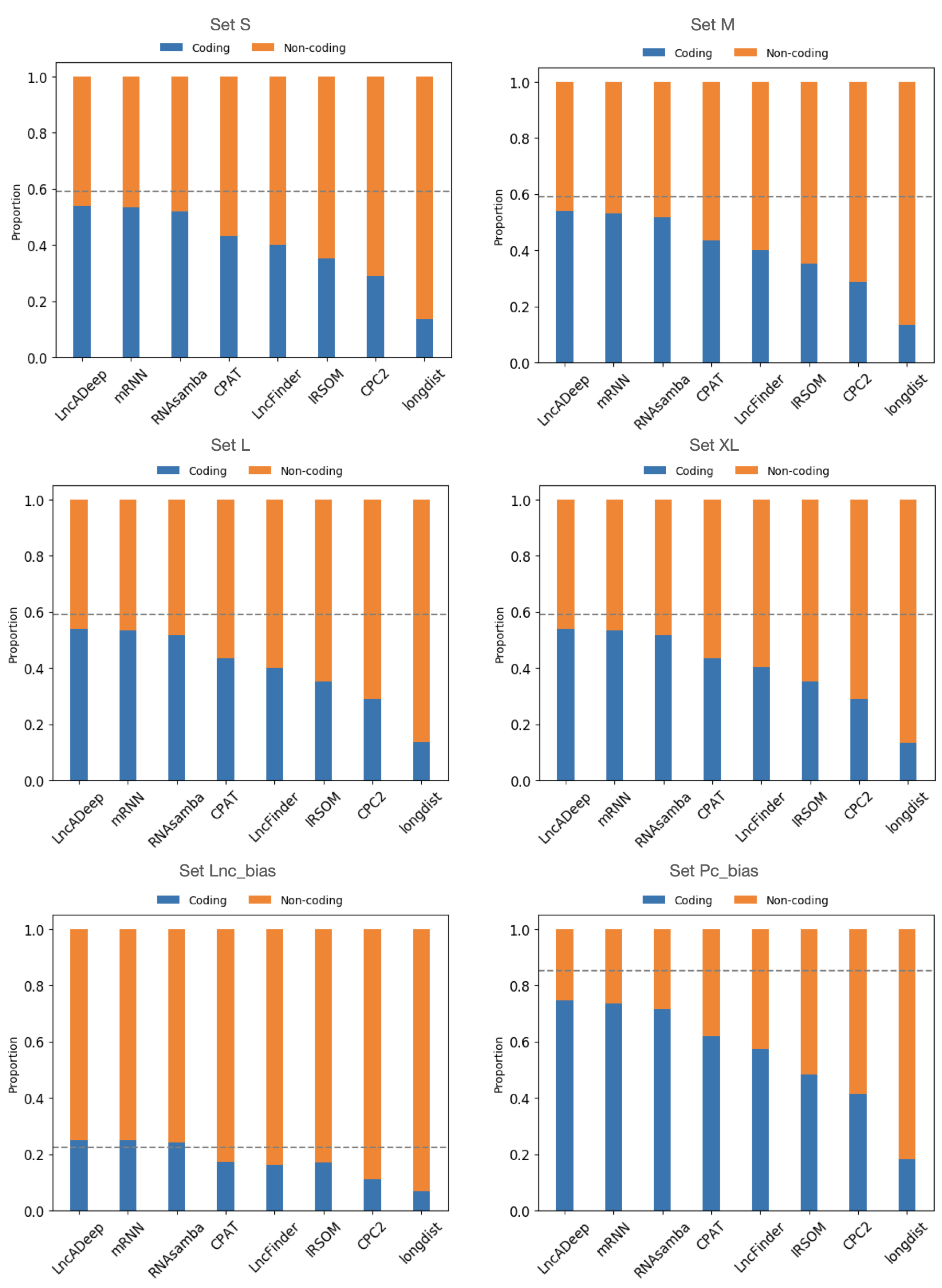


Supplementary Figure 3: Proportions of predicted coding (mRNA) and non-coding (lncRNA) transcripts for all test sets S,M,L,XL, Lnc_bias and Pc_bias. The proportion of predicted non-coding and protein-coding transcripts is marked in orange and blue, respectively. The true proportions for each test set are indicated with horizontal dashed lines.

Precision-recall curves for reference test set Lnc_bias when evaluating prediction of lncRNAs


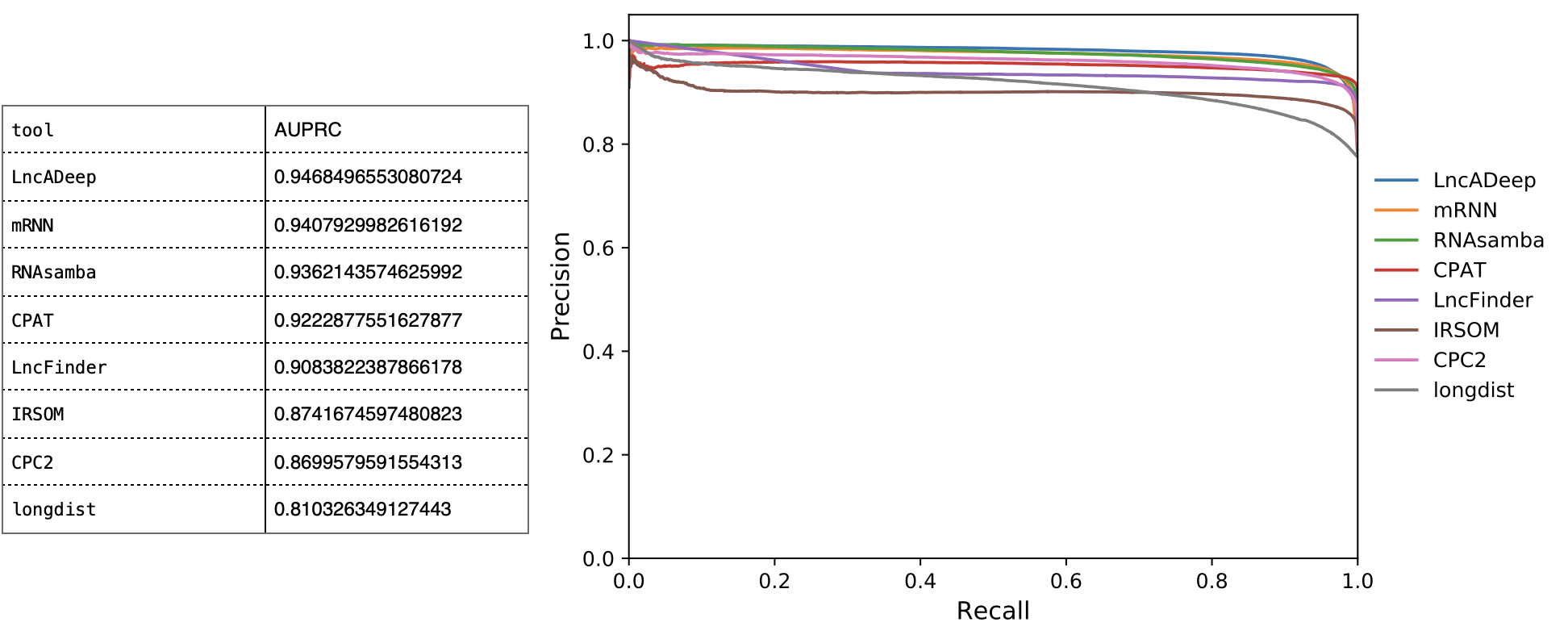


Supplementary Figure 4: Precision-recall curves and area under precision recall curve (AUPRC) for all tested tools on the Lnc_bias test set (left) and Pc_bias test set (right) when prediction classes have been reversed. The curves were drawn from reversed prediction probabilities (1-probability), requiring the tools thus to predict for the presence of lncRNAs instead of mRNAs. Precision and recall do not take into account True negative -predictions, thus in Figure1, true predictions of lncRNAs remain un accounted for, and here true predictions of mRNAs remain unaccounted for.
